# Evaluating the quality and reliability of radiation dermatitis related videos on Douyin and Bilibili: a cross-sectional analysis

**DOI:** 10.3389/fpubh.2026.1851530

**Published:** 2026-07-27

**Authors:** Peiran Wang, Shuang Yu, Ning Li, Xinrui Hu, Ning Du, Xinqi Liu, Aiping Tian

**Affiliations:** National Cancer Center/National Clinical Research Center for Cancer/Cancer Hospital, Chinese Academy of Medical Sciences and Peking Union Medical College, Beijing, China

**Keywords:** health information dissemination, radiation dermatitis (RD), short-form video platforms, user interaction, video quality

## Abstract

**Background:**

Radiation dermatitis (RD), a common adverse reaction to radiotherapy (RT), is widely discussed on video-sharing platforms. However, user-generated content about RD lacks systematic scientific validation. This study evaluates the reliability and quality of RD-related videos on two major social media platforms: Douyin and Bilibili.

**Methods:**

We conducted a cross-sectional study on Douyin and Bilibili, using keywords including “radiation dermatitis (放射性皮炎),” “radiotherapy-induced dermatitis (放疗诱导性皮炎),” “radiation skin injury (放射性皮肤损伤),” and “radiation dermatitis care (放射性皮炎护理)”. A total of 190 eligible short videos were included for analysis. We collected data on video characteristics, including title, uploader identity, upload date, video duration, content type, engagement metrics (likes, comments, shares, saves), presentation format, and quality scores. Video quality and reliability were assessed using the modified DISCERN (mDISCERN), the Journal of the American Medical Association (JAMA) benchmark criteria, and the Global Quality Scale (GQS) by two independent reviewers.

**Results:**

The analysis revealed that Douyin videos were significantly shorter in duration than those on Bilibili (*p* < 0.05), yet they demonstrated higher audience engagement. Douyin yielded significantly higher median mDISCERN and JAMA scores than Bilibili, while the between-group difference in GQS scores did not reach statistical significance. Videos created by professional healthcare providers demonstrated higher reliability and quality, with mDISCERN scores of 3 and GQS scores of 3. Correlation analysis revealed a strong significant positive correlation between video shares and saves. Engagement metrics showed positive correlations with all three quality scores but with gradient differences in strength: strong correlations with mDISCERN scores, moderate correlations with GQS scores, and weak correlations with JAMA scores.

**Conclusions:**

Social media platforms support the dissemination of RD-related health information to some extent, but the overall quality of videos remains suboptimal. We recommend that professional healthcare providers obtain official platform certification to promote the spread of high-quality RD-related content.

## Introduction

Radiation dermatitis (RD) represents a prevalent adverse event associated with radiotherapy (RT), and the vast majority of patients receiving RT develop skin reactions of varying severity (1–3). The incidence and severity of RD are influenced by multiple clinical factors, including tumor site, radiation delivery technique, and total prescribed dose, among others (4–7).

Most patients present with mild erythema and dry desquamation, while only a small proportion progress to severe exudative ulceration and necrosis. Severe high-grade RD may occasionally necessitate temporary RT interruption, which not only increases healthcare expenditure but also markedly impairs patients' quality of life. A considerable proportion of affected patients experience psychological distress, including anxiety and sleep disturbances (8–11). Standardized skin care protocols combined with structured patient education can effectively reduce the risk of moderate-to-severe RD (12–14).

Short-video platforms have emerged as a major channel for public health information acquisition amid the global digitalization trend (15–18). Compared with traditional text-based content, video health communication delivers information more concisely and intuitively, and can effectively drive positive health behaviors (19). Skincare-related content on TikTok has accumulated hundreds of millions of views globally, reflecting broad public attention to skin health knowledge (20, 21). However, the unregulated content creation ecosystem on social media raises concerns about the reliability of medical information (22). Misleading health content may lead to inappropriate medical decisions, underscoring the necessity of systematic quality assessment of online health videos.

In China, Douyin (the domestic version of TikTok) and Bilibili are the two dominant short-video platforms, with hundreds of millions of monthly active users and broad coverage of health-related content (23, 24). Existing studies have evaluated video quality across multiple disease areas on these platforms: liver cancer and inflammatory bowel disease content generally shows poor quality (25, 26), while cosmetic surgery-related videos achieve satisfactory reliability (27). Nevertheless, the quality and reliability of RD-related short videos on these platforms remain unexplored.

To address this research gap, we conducted a cross-sectional analysis to systematically evaluate the content quality, information reliability, and user engagement of RD-related videos on Douyin and Bilibili. This study aims to provide empirical references for standardizing medical short-video content and improving public access to evidence-based RD health education.

## Materials and methods

### Ethical considerations

All information was sourced from publicly available videos on Douyin and Bilibili. Since the data does not involve any personal acknowledgments privacy-related content, ethical review was not required.

### Video collection

On November 1, 2025, video retrieval was conducted separately on Bilibili and Douyin using four pre-defined Chinese keywords: “radiation dermatitis (放射性皮炎),” “radiotherapy-induced dermatitis (放疗诱导性皮炎),” “radiation skin injury (放射性皮肤损伤),” “radiation dermatitis care (放射性皮炎护理)”. As the standard diagnostic term in clinical practice, radiation dermatitis is universally accepted by medical professionals and familiar to the general public, serving as the most precise and representative core keyword; the supplementary synonymous terms cover common alternative expressions across different content scenarios, effectively minimizing the risk of missing eligible videos due to inconsistent terminology. Identical search terms and screening rules were applied to both platforms to ensure the comparability of samples from Douyin and Bilibili.

**Figure 1:**
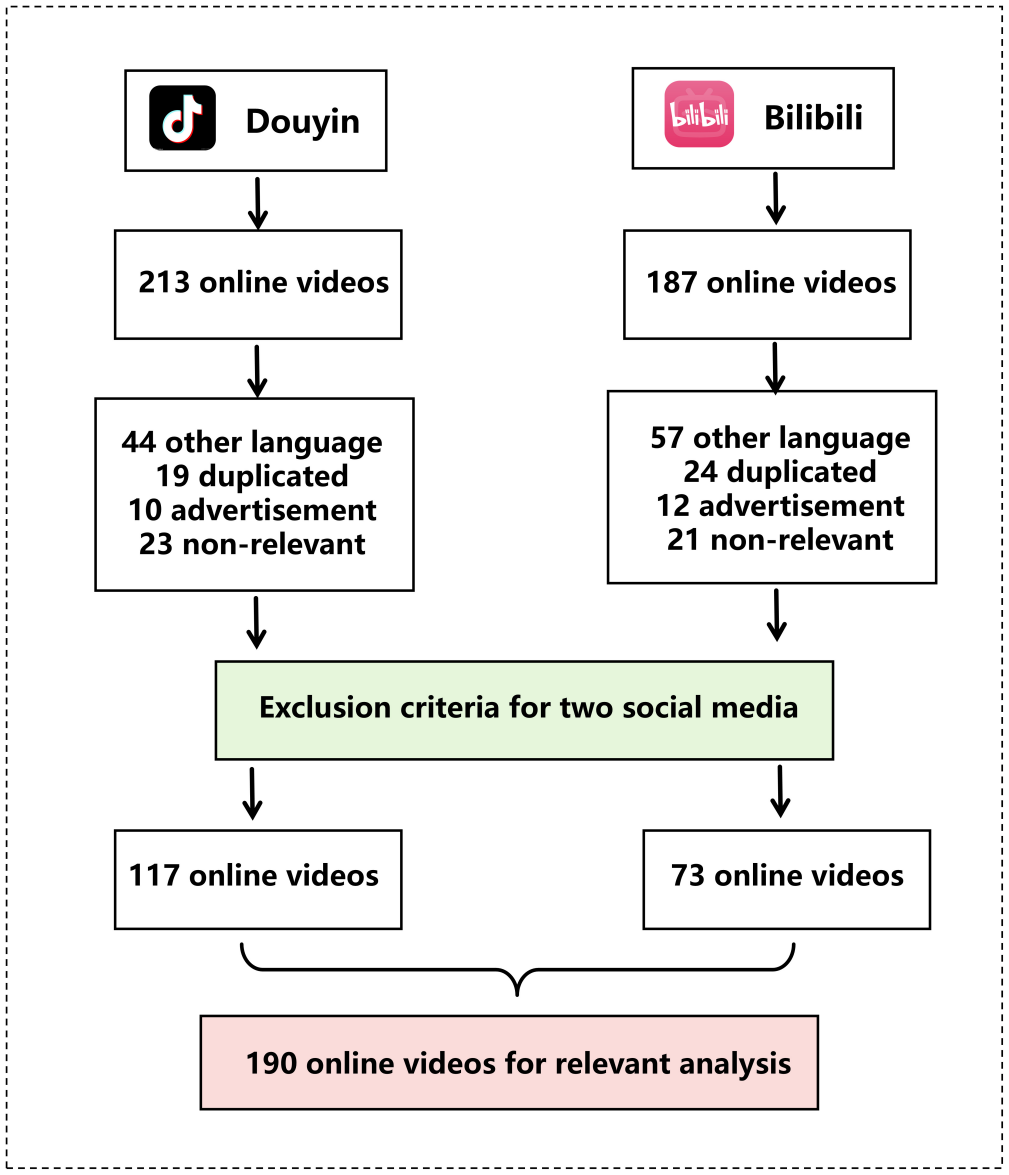
Flowchart of the video screening and inclusion process of radiation dermatitis. Numbers of videos excluded at each stage and corresponding reasons are presented in detail.

Prior to conducting the search, we logged out of all accounts and cleared search histories to avoid bias resulting from personalized recommendations. Video screening and quality scoring were independently completed by two trained licensed physicians (Jingsong Wang, Bingxi Liu) with master's degrees. Both evaluators received standardized training on inclusion/exclusion criteria and scoring rules prior to formal assessment. The inter-rater agreement was assessed using Cohen's κ coefficient. The κ values for video inclusion screening, GQS, JAMA, and mDISCERN assessments were 0.91, 0.84, 0.86, and 0.82, respectively, indicating excellent agreement between the two reviewers. Any discrepancies between the two evaluators were resolved through discussion or final adjudication by the corresponding author. The exclusion criteria were as follows: (1) videos unrelated to the topic; (2) duplicate videos; (3) videos used for advertising or commercial purposes; (4) videos with other language. Additionally, all search results were limited to independent short-video content, while livestream recordings, image-based posts, and pure text content were excluded prior to formal screening to strictly align with the study's research scope. Following the application of all predefined exclusion criteria, 101 non-target language videos, 43 duplicates, 22 advertisements, and 44 irrelevant videos were removed, leaving 190 eligible videos for final analysis. For each included video, baseline metadata (URL, uploader, likes, saves, shares, comments, and duration) was extracted and documented in a Microsoft Excel spreadsheet. The screening process is summarized in Figure 1.

This study collected and analyzed a total of 190 videos from Douyin and Bilibili. We systematically extracted and recorded various video characteristics, including view counts, likes, comments, saves, shares, duration, upload date, upload source, video content, GQS, JAMA, and mDISCERN scores. The duration of each video was measured using the platform's built-in video player, which displayed the total video duration (in seconds). Based on the uploader's account name, verification status, and information within the video, videos were categorized into four main groups: professional healthcare providers, non-professional healthcare providers, institutions, and individual users.

This study employed three commonly used standardized scales to evaluate video content: GQS, JAMA, and mDISCERN. These scales were used to assess and analyze the quality and effectiveness of the videos. GQS is a widely used tool for evaluating the quality of health information in videos, assessing dimensions such as quality, fluency, comprehensiveness, and patient utility (Supplementary Table 1). It uses a 5-point scale ranging from 1 (poor quality) to 5 (excellent fluency and quality). The JAMA score assesses the reliability of videos based on four key criteria: (1) disclosure of author identity; (2) copyright information, reference lists, and content sources; (3) provision of the initial date and subsequent updates; and (4) disclosure of conflicts of interest, funding, sponsorship, advertising support, and video ownership (Supplementary Table 2). The total score is 4 points, with 1 point awarded for each criterion. The mDISCERN is a widely validated and applied tool that evaluates videos based on five questions: (1) Is the video clear, concise, and easy to understand? (2) Are the sources of this information reliable? (3) Is the information presented balanced and fair? (4) Does it provide patients with additional sources of information? (5) Are areas of uncertainty or controversy appropriately addressed? A score of 1 is assigned for each affirmative answer, and 0 for each negative answer (Supplementary Table 3). The aforementioned tools have been validated in previous studies and are particularly suitable for research related to social media platforms. Additionally, we evaluated the video content, which was categorized into seven types: clinical manifestations, etiology and risk factors, news reports, patient experiences or other, preventive measures, scientific introductions, and treatment methods.

### Statistical analysis

Data analysis was performed using IBM SPSS Statistics 26.0 for Windows and R version 4.3.1. The Shapiro–Wilk test was used to assess the normality of continuous variables. Continuous variables that followed a normal distribution were expressed as “mean ± standard deviation (mean ± SD),” while those that did not follow a normal distribution were expressed as “median, minimum–maximum, and 25th−75th percentiles [M (P25, P75)].” The Cohen's κ coefficient was used to quantify inter-rater reliability between two raters, with the following criteria: κ > 0.8 indicates excellent reliability; 0.6 <κ ≤ 0.8 indicates good reliability; 0.4 <κ ≤ 0.6 indicates moderate reliability; and κ ≤ 0.4 indicates poor reliability. The Mann-Whitney U test was used for comparisons between two groups, and the Kruskal-Wallis H test was adopted for comparisons across four uploader subgroups and seven content categories. For *post hoc* pairwise intergroup comparisons, Dunn's test with Bonferroni correction for multiple comparisons was further performed to identify specific pairs exhibiting significant differences. The significance threshold was set at *P* < 0.05. Spearman's correlation analysis was employed to reveal correlations among different video variables. Given the non-normal distribution of our data, *P* < 0.05 was considered statistically significant. Data visualization was performed using GraphPad Prism 8.0.1 for Windows.

## Results

### Overview of the video screening process

A systematic search using a predefined keyword set centered on radiation dermatitis on Douyin and Bilibili initially identified 400 videos. The video pool was subsequently optimized through a rigorous selection process, removing 101 non-target language videos (44 from Douyin and 57 from Bilibili), 43 duplicates (19 from Douyin and 24 from Bilibili), 22 advertisements (10 from Douyin and 12 from Bilibili), and 44 irrelevant videos (23 from Douyin and 21 from Bilibili), resulting in 190 eligible videos included for further analysis.

### Content analysis of online videos

**Figure 2:**
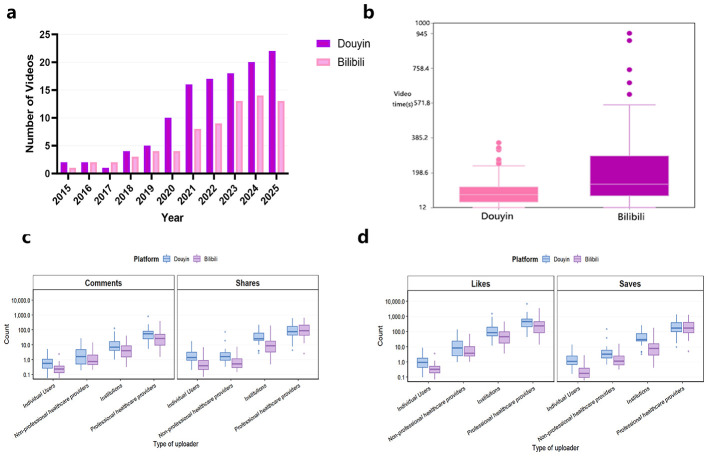
Characteristics of selected videos from Bilibili and Douyin. **(a)** Upload time of the included online videos. **(b)** Video duration comparison between Douyin and Bilibili. **(c)** Number of comments and shares for different types of video content. **(d)** Number of likes and saves for different types of video content.

The selected online videos were uploaded between April 13, 2015, and November 1, 2025 (Figure 2a). For Douyin, the annual distribution of included videos from 2015 to 2025 was as follows: 2 videos in 2015 (2%), 2 videos in 2016 (2%), 1 video in 2017 (1%), 4 videos in 2018 (3%), 5 videos in 2019 (4%), 10 videos in 2020 (9%), 16 videos in 2021 (14%), 17 videos in 2022 (15%), 18 videos in 2023 (15%), 20 videos in 2024 (17%), and 22 videos in 2025 (18%). For Bilibili, the corresponding numbers were 1 video in 2015 (1%), 2 videos in 2016 (3%), 2 videos in 2017 (3%), 3 videos in 2018 (4%), 4 videos in 2019 (5%), 4 videos in 2020 (5%), 8 videos in 2021 (11%), 9 videos in 2022 (12%), 13 videos in 2023 (18%), 14 videos in 2024 (19%), and 13 videos in 2025 (18%).

The median duration of RD videos was 97 (12, 359) s on Douyin and 202 (40, 945) s on Bilibili, with a statistically significant difference between the two platforms (*P* < 0.001) (Figure 2b), which may be attributed to differences in platform content length policies. Furthermore, videos were categorized into four uploader groups: professional healthcare providers, non-professional healthcare providers, individual users, and institutions. Across all four engagement metrics (likes, saves, comments, and shares), Douyin showed significantly higher values than Bilibili. As indicated by the *P*-values in Figures 2c, d, significant differences in engagement levels across uploader categories were observed for some subgroups but not for others.

### Video uploaders

**Figure 3:**
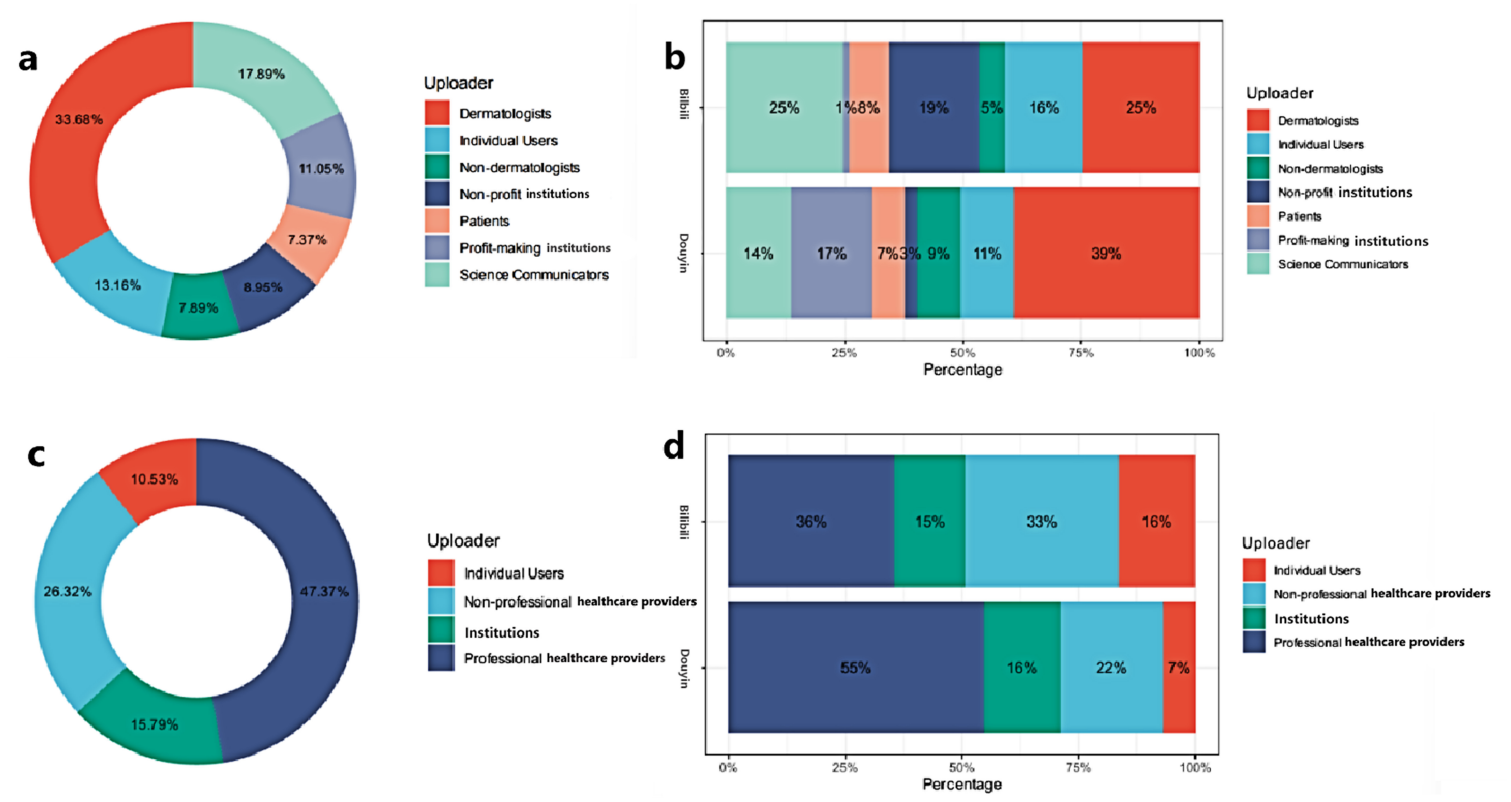
Uploader category distribution of radiation dermatitis-related short videos. Top row **(a, b)**: Detailed seven-subgroup creator classification. **(a)** Overall proportion donut chart; **(b)** Platform-stratified stacked percentage bar chart (Douyin vs Bilibili). Bottom row **(c, d)**: Simplified four merged creator categories aggregated from the seven subgroups. **(c)** Overall donut chart of four merged groups; **(d)** Platform-stratified stacked bar chart of merged categories. Category aggregation mapping:Individual Users (light orange): Individual Users and Patients; Non-professional healthcare providers (light blue): Science Communicators; Institutions (light green): Non-profit and Profit-making Institutions; Professional healthcare providers (dark blue): Dermatologists and Non-dermatologists.

In this study, video uploaders were primarily categorized as dermatologists (33.68%), non-dermatologists (7.89%), science communicators (17.89%), individual users (13.16%), for-profit institutions (11.05%), non-profit institutions (8.95%), and patients (7.37%) (Figure 3a). On Bilibili, dermatologists and science communicators accounted for the largest shares, both at 25%, followed by individual users, while for-profit institutions and non-dermatologists had lower shares. In contrast, on Douyin, dermatologists were the primary uploaders, accounting for 39%, followed by for-profit institutions and science communicators, with individual users contributing less (Figure 3b). Professional healthcare providers uploaded 47.37% of the videos, while others accounted for 52.63% (Figure 3c). On Bilibili, professional healthcare providers contributed 36% of the content, while individual users accounted for the smallest share at 16%. Similarly, on Douyin, professional healthcare providers accounted for 55% and individual users for 7% (Figure 3d). This highlights that professional healthcare providers are the primary content providers on both platforms, particularly on Douyin (Supplementary Table 4).

### Video content

**Figure 4:**
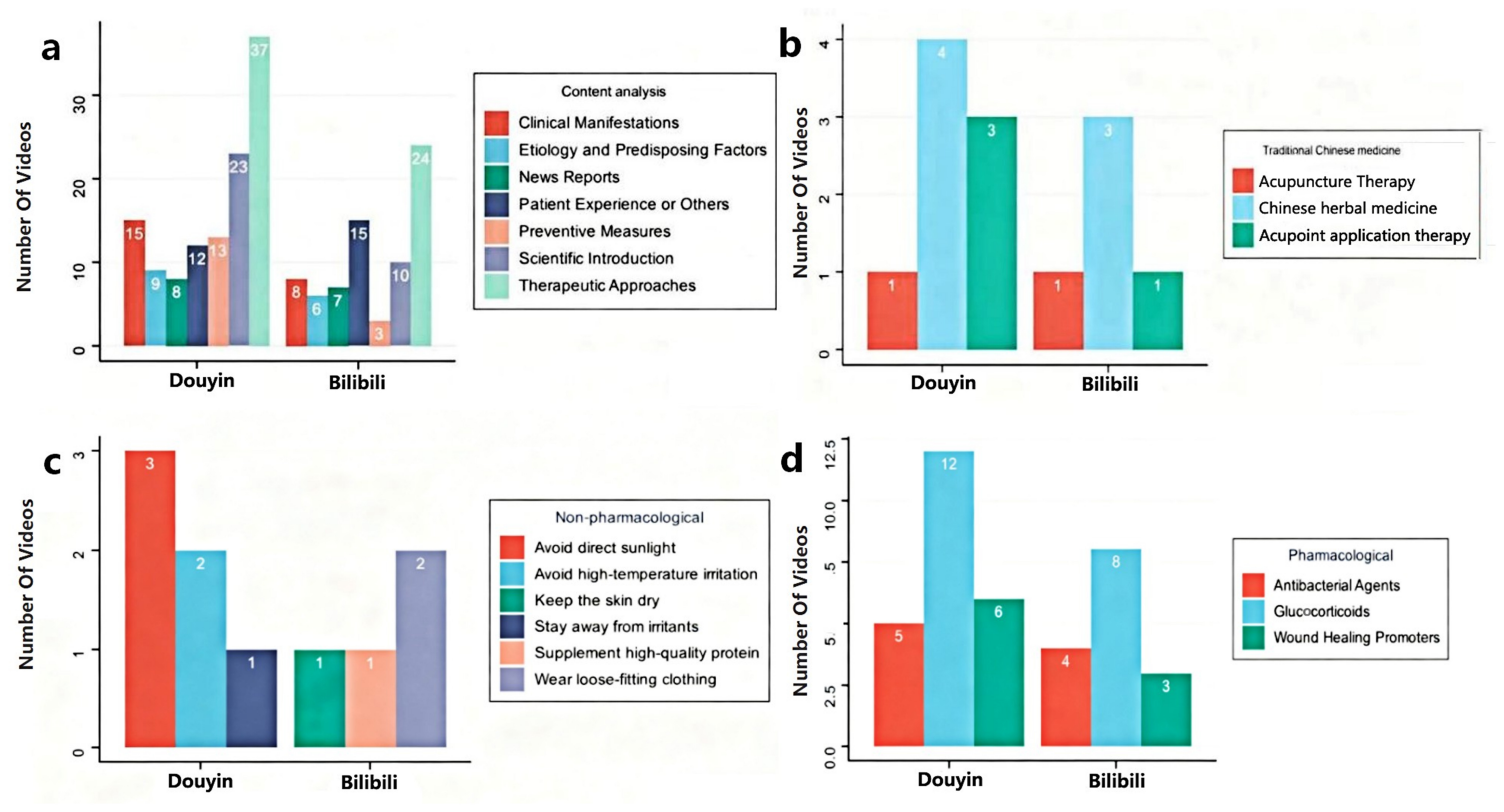
Comparative classification statistics of radiation dermatitis (RD)-related videos from Douyin and Bilibili. **(a)** Distribution of seven major RD content categories stratified by platform (Douyin, *n* = 117; Bilibili, *n* = 73). Numerical values above each bar represent the exact number of videos in each subgroup, and the aggregate total count of each category is summarized in Table 1. **(b)** Subtypes of traditional Chinese medicine intervention content covered in included videos. **(c)** Classification of non-pharmacological skin care advice for radiation dermatitis. **(d)** Classification of pharmacological intervention schemes for radiation dermatitis.

In the video content analysis section, the content was categorized into seven categories: clinical manifestations (12.1%), etiology and risk factors (7.9%), news reports (7.9%), patient experiences or other (14.2%), preventive measures (8.4%), scientific introductions (17.4%), and treatment methods (32.1%). Among all 190 videos, 117 were from Douyin and 73 from Bilibili. The seven content categories were distributed as follows: Clinical Manifestations (15 Douyin, 8 Bilibili, total *n* = 23); Etiology and Predisposing Factors (9, 6, *n* = 15); News Reports (8, 7, *n* = 15); Patient Experience or Others (12, 15, *n* = 27); Preventive Measures (13, 3, *n* = 16); Scientific Introduction (23, 10, *n* = 33); Therapeutic Approaches (37, 24, *n* = 61) (Figure 4a, Table 1). We compared the basic content categories of videos on Douyin and Bilibili: on Douyin, the top three categories by proportion were treatment methods for RD (37/117, 32%), followed by scientific introductions (23/117, 20%) and clinical manifestations (15/117, 13%) (Supplementary Table 5). A similar distribution of video content was observed on Bilibili, with most videos also focusing on treatment methods (24/73, 33%), followed by patient experience or others topics (15/73, 21%) and scientific introductions (10/73, 14%). Among treatment methods, Traditional Chinese Medicine (TCM)-related content—including acupuncture, herbal medicine, and acupoint plaster application—was covered in 13 videos. On Douyin, herbal medicine was mentioned 4 times and acupoint plaster application 3 times; on Bilibili, herbal medicine was mentioned 3 times, while acupuncture and acupoint plaster application were each mentioned once. The proportion of TCM-related content on Douyin was higher than on Bilibili (Figure 4b). Non-pharmacological recommendations included avoiding direct sunlight, avoiding high-temperature exposure, keeping the skin dry, staying away from irritants, wearing loose-fitting clothing, and consuming high-quality protein. On Douyin, avoiding direct sunlight was mentioned 3 times and avoiding high-temperature exposure 2 times, with higher mention frequencies than on Bilibili; on Bilibili, categories such as loose-fitting clothing were mentioned relatively less frequently (Figure 4c). Regarding medication, 38 videos covered drug-related content (antimicrobials, corticosteroids, and wound healing promoters). Twenty videos recommended corticosteroids, 9 recommended antimicrobials, and 9 mentioned wound healing promoters. The proportion of drug-related content on Douyin was higher than on Bilibili (Figure 4d).

**Table 1:** Comparison of video characteristics, engagement metrics, and quality scores across different content categories of radiation dermatitis–related short videos.

Variables	Total (*n* = 190)	Clinical manifestations (*n* = 23)	Etiology and predisposing factors (*n* = 15)	News reports (*n* = 15)	Patient experience or others (*n* = 27)	Preventive measures (*n* = 16)	Scientific introduction (*n* = 33)	Therapeutic approaches (*n* = 61)	Statistic	*P*
Video duration (seconds), M (Q1, Q3)	94.00 (57.00, 172.75)	104.00 (52.00, 178.00)	141.00 (71.00, 262.00)	78.00 (54.50, 105.00)	85.00 (59.00, 151.50)	86.00 (40.25, 110.25)	125.00 (51.00, 193.00)	88.00 (57.00, 199.00)	H=5.61#	0.468
Likes, M (Q1, Q3)	75.50 (9.25, 631.00)	90.00 (10.00, 838.50)	90.00 (50.50, 613.50)	66.00 (12.50, 100.00)	23.00 (1.00, 120.50)	36.00 (7.75, 112.75)	120.00 (24.00, 1,411.00)	127.00 (14.00, 1,138.00)	H = 12.14#	0.059
Saves, M (Q1, Q3)	26.50 (3.00, 342.75)	30.00 (3.00, 406.00)	25.00 (8.00, 144.00)	16.00 (5.50, 25.00)	22.00 (0.50, 138.50)	12.00 (1.75, 30.00)	34.00 (5.00, 608.00)	63.00 (3.00, 747.00)	H = 8.83#	0.183
Comments, M (Q1, Q3)	9.00 (1.00, 70.00)	14.00 (1.00, 177.00)	12.00 (0.50, 26.50)	8.00 (1.00, 82.50)	0.00 (0.00, 15.50)	3.00 (0.75, 5.25)	15.00 (5.00, 241.00)	9.00 (1.00, 91.00)	H = 14.67#	0.023
Shares, M (Q1, Q3)	13.00 (1.00, 151.75)	19.00 (1.50, 465.00)	14.00 (5.50, 220.00)	6.00 (0.00, 31.00)	9.00 (1.00, 67.50)	7.00 (1.00, 22.50)	18.00 (1.00, 115.00)	54.00 (3.00, 282.00)	H = 7.89#	0.246
mDISCERN score, M (Q1, Q3)	2.00 (2.00, 3.00)	2.00 (2.00, 3.00)	3.00 (2.00, 3.00)	2.00 (1.50, 2.00)	2.00 (1.00, 2.50)	2.00 (1.00, 2.25)	3.00 (2.00, 3.00)	3.00 (2.00, 3.00)	H = 18.80#	0.005
GQS score, M (Q1, Q3)	3.00 (2.00, 3.00)	3.00 (2.00, 3.00)	3.00 (3.00, 3.00)	2.00 (2.00, 3.00)	2.00 (2.00, 3.00)	2.00 (2.00, 2.25)	3.00 (2.00, 3.00)	3.00 (2.00, 3.00)	H = 15.38#	0.018
JAMA score, M (Q1, Q3)	3.00 (2.00, 3.00)	2.00 (2.00, 3.00)	2.00 (2.00, 3.00)	2.00 (2.00, 2.00)	2.00 (2.00, 3.00)	3.00 (2.00, 3.00)	2.00 (2.00, 3.00)	3.00 (2.00, 3.00)	H = 15.68#	0.016

#: Kruskal-Wallis H test for overall between-group differences; *post hoc* pairwise comparisons were conducted via Dunn's test with Bonferroni multiple testing correction. Superscript letters denote significant pairwise differences (*P* < 0.05); groups sharing identical letters show no statistical discrepancy.

M: Median, Q1: 1st Quartile, Q3: 3rd Quartile.

### Video quality and reliability

**Figure 5:**
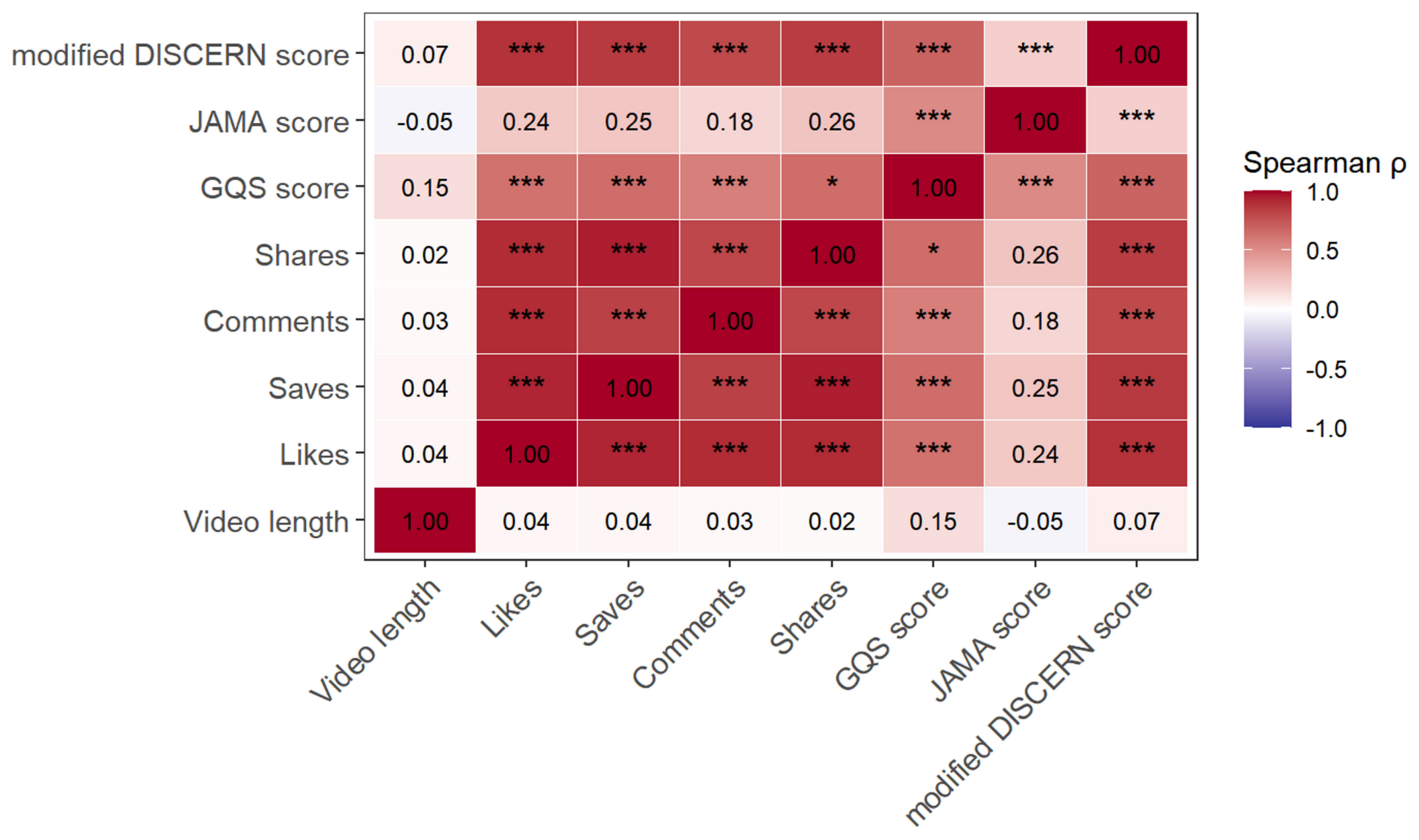
Spearman correlation matrix for radiation dermatitis video variables (mDISCERN, JAMA, and GQS scores). * *P* < 0.05; ** *P* < 0.01; *** *P* < 0.001.

A comparison of video quality and reliability among uploaders of different types revealed significant differences in GQS, JAMA, and mDISCERN scores, as shown in the Figure 5. Professional healthcare providers scored significantly higher on the GQS, JAMA, and mDISCERN scales than institutions, non-professional healthcare providers, and individual users.

Differences in video quality scores [modified DISCERN (mDISCERN), JAMA benchmark criteria, and Global Quality Scale (GQS)] between the Douyin and Bilibili platforms were compared using the Mann-Whitney U test, given the non-normal distribution of all scoring variables. Detailed results are presented in Table 2. After applying the predefined inclusion and exclusion criteria, a total of 190 eligible videos were included in the final analysis, comprising 117 videos from Douyin and 73 videos from Bilibili.

**Table 2:** Comparison of video characteristics, engagement metrics, and quality scores between the douyin and bilibili platforms.

Variables	Total (*n* = 190)	Bilibili (*n* = 73)	Douyin (*n* = 117)	Statistic	*P*
Video duration (seconds), M (Q1, Q3)	94.00 (57.00, 172.75)	144.00 (75.00, 618.00)	81.00 (44.00, 124.00)	Z = −4.56	**<0.001**
Likes, M (Q1, Q3)	75.50 (9.25, 631.00)	7.00 (1.00, 127.00)	121.00 (49.00, 1,710.00)	Z = −6.49	**<0.001**
Saves, M (Q1, Q3)	26.50 (3.00, 342.75)	7.00 (1.00, 96.00)	40.00 (7.00, 682.00)	Z = −4.20	**<0.001**
Comments, M (Q1, Q3)	9.00 (1.00, 70.00)	0.00 (0.00, 11.00)	17.00 (4.00, 185.00)	Z = −6.59	**<0.001**
Shares, M (Q1, Q3)	13.00 (1.00, 151.75)	3.00 (0.00, 22.00)	36.00 (5.00, 529.00)	Z = −5.35	**<0.001**
mDISCERN score, M (Q1, Q3)	2.00 (2.00, 3.00)	2.00 (1.00, 3.00)	3.00 (2.00, 3.00)	Z = −5.31	**<0.001**
GQS score, M (Q1, Q3)	3.00 (2.00, 3.00)	2.00 (2.00, 3.00)	3.00 (2.00, 3.00)	Z = −1.45	0.148
JAMA score, M (Q1, Q3)	3.00 (2.00, 3.00)	2.00 (2.00, 3.00)	3.00 (2.00, 3.00)	Z = −3.03	**0.002**

Z: Mann-Whitney test.

M: Median, Q1: 1st Quartile, Q3: 3rd Quartile.

Between-platform comparisons revealed that Douyin had significantly higher median mDISCERN scores (*P* < 0.001) and JAMA scores (*P* = 0.002) than Bilibili. For GQS scores, the median value was one point higher in the Douyin group than in the Bilibili group; however, the difference did not reach statistical significance (*P* = 0.148), indicating a potential trend toward higher overall educational quality in Douyin videos.

Video characteristics on both platforms were analyzed, including video duration and audience engagement (likes, comments, saves, and shares). Analysis of Table 2 revealed significant differences between Douyin and Bilibili in these aspects (*p* < 0.001), with Douyin videos being significantly shorter than those on Bilibili. Douyin videos demonstrated higher audience engagement, receiving the highest number of likes (median: 121, IQR: 40–1,710), comments (median: 17, IQR: 4–185), saves (median: 40, IQR: 7–682), and shares (median: 36, IQR: 5–529), while Bilibili videos showed lower values across all engagement metrics.

Table 2 provides a detailed analysis of video characteristics based on uploader identity, and all of these characteristics show significant differences. Specifically, 20 videos (10.53%) were posted by individual users, 50 (26.32%) by non-professional healthcare providers, 30 (15.79%) by institutions, and 90 (47.37%) by professional healthcare providers. Videos uploaded by professional healthcare providers were significantly longer than those from other groups, and they also performed exceptionally well in terms of engagement metrics, whereas videos uploaded by individual users performed poorly in these aspects. Analysis of video duration showed that the median length of videos posted by individual users was 62 (IQR: 41.5–105), while that of non-professional healthcare providers was 91.5 (IQR: 57–195). For institutions it was 86 (IQR: 64.75–187), and for professional healthcare providers it was 105.5 (IQR: 58.25–191.25). The central tendency and dispersion of video durations were relatively similar across all groups. Regarding audience engagement metrics, there were extremely significant differences among the various upload groups (all *P*-values <0.001). In terms of the number of likes, videos posted by professional healthcare providers received the highest number of likes, while those posted by individual users received the lowest.

In terms of video quality scores, the differences among uploaders were also extremely significant (all *P*-values <0.001). The median mDISCERN score for videos posted by professional healthcare providers was 3.00 (IQR: 2.00–3.00), while that for videos posted by individual users was only 1.00 (IQR: 1.00–2.00). Overall, videos posted by professional healthcare providers significantly outperformed those from other uploaders in both audience engagement and quality scores, while videos posted by individual users ranked lowest in both engagement metrics and quality scores. Non-professional healthcare providers and institutions performed somewhere in between (Table 3).

**Table 3:** Comparison of video characteristics, engagement metrics, and quality scores across different uploader categories.

Variables	Total (*n* = 190)	Individual Users (*n* = 20)	Non-professional healthcare providers (*n* = 50)	Institutions (*n* = 30)	professional healthcare providers (*n* = 90)	Statistic	*P*
Video duration(seconds), M (Q1, Q3)	94.00 (57.00, 172.75)	62.00 (41.50,105.00)	91.50 (57.00, 195.00)	86.00 (64.75, 187.00)	105.50 (58.25, 191.25)	H = 4.07#	0.254
Likes, M (Q1, Q3)	75.50 (9.25, 631.00)	1.00 (0.00, 65.25)	11.00 (2.00, 33.75)	97.50 (12.00, 303.50)	372.00 (84.50, 2,108.25)	H = 74.93#	**<0.001**
Saves, M (Q1, Q3)	26.50 (3.00, 342.75)	1.00 (0.00, 21.00)	3.00 (2.00, 9.50)	23.50 (4.00, 149.25)	224.00 (30.25, 1,173.50)	H = 74.39#	**<0.001**
Comments, M (Q1, Q3)	9.00 (1.00, 70.00)	0.00 (0.00, 8.25)	1.50 (0.00, 7.75)	5.50 (1.00, 142.25)	33.00 (6.50, 289.00)	H = 49.45#	**<0.001**
Shares, M (Q1, Q3)	13.00 (1.00, 151.75)	1.00 (0.00, 31.00)	1.00 (0.00, 7.00)	14.00 (1.25, 171.25)	83.50 (16.25, 595.50)	H = 69.24#	**<0.001**
mDISCERN score, M(Q1, Q3)	2.00 (2.00, 3.00)	1.00 (1.00, 2.00)	2.00 (1.00, 2.00)	2.00 (2.00, 3.00)	3.00 (2.00, 3.00)	H = 62.45#	**<0.001**
GQS score, M (Q1, Q3)	3.00 (2.00, 3.00)	2.00 (2.00, 2.00)	2.00 (2.00, 2.00)	3.00 (2.00, 3.00)	3.00 (3.00, 3.00)	H = 50.51#	**<0.001**
JAMA score, M (Q1, Q3)	3.00 (2.00, 3.00)	2.00 (2.00, 2.00)	2.00 (2.00, 3.00)	2.00 (2.00, 2.00)	3.00 (3.00, 3.00)	H = 65.46#	**<0.001**

#: Kruskal-Wallis H test for overall between-group differences; *post hoc* pairwise comparisons were conducted via Dunn's test with Bonferroni multiple testing correction. Superscript letters denote significant pairwise differences (*P* < 0.05); groups sharing identical letters show no statistical discrepancy. M: Median, Q1: 1st Quartile, Q3: 3rd Quartile.

### Correlation analysis

Spearman's rank correlation analysis was applied given the non-normal data distribution, to examine associations between engagement metrics (likes, comments, saves, shares) and the three quality assessment scales (GQS, JAMA, mDISCERN). All four engagement metrics were strongly positively correlated with one another, with the strongest association detected between shares and saves (ρ = 0.95, *P* < 0.001). Correlations between engagement metrics and quality scores differed markedly across assessment tools: Modified DISCERN scores showed strong, statistically significant positive correlations with all engagement indicators (ρ = 0.80–0.89, all *P* < 0.001). GQS scores displayed moderate significant positive correlations with engagement metrics (ρ = 0.57–0.65, all *P* < 0.05). By contrast, JAMA scores only exhibited weak positive linear trends against engagement metrics (ρ = 0.18–0.26), yet none of these weak associations reached statistical significance (all *P* > 0.05). Video length showed negligible, non-significant correlations with all engagement and quality variables (|ρ| <0.15). Full Spearman correlation coefficients and significance markers are visualized in Figure 5.

## Discussion

Radio dermatitis is a common adverse reaction to radiotherapy (RT), and standardized patient education is crucial for reducing moderate-to-severe cutaneous complications (28). In clinical practice, high-quality health education enables patients to identify risk factors at an early stage, master preventive measures, and properly manage early skin lesions, thereby effectively lowering the risk of moderate-to-severe radiodermatitis (29). However, limited by uneven distribution of medical resources and insufficient outpatient consultation time, most radiotherapy patients cannot obtain systematic and comprehensive skin care guidance from clinical medical staff. With the rapid development of digital health, surveys show that as many as 87% of the public take social media as their top source of health information, and 31.7%−41% of short-video users prefer watching health popular science content (30). To date, no studies have systematically evaluated the quality of short videos related to radiodermatitis. Existing relevant research mostly focuses on other diseases such as gastric cancer, laryngeal cancer and thyroid disorders, and generally points out that health popular science content on social media varies widely in quality (31, 32). Given the high incidence and specialized nursing requirements of radiodermatitis, a multidimensional analysis of relevant popular science content on mainstream short-video platforms is urgently needed. Accordingly, this study systematically collected 190 radiodermatitis-related short videos from Douyin and Bilibili and performed quality assessment. This research aims to provide references for the public to identify high-quality health information, offer suggestions for creators to optimize health communication formats.

### Discussion of video engagement metrics

The significant difference in engagement between the two platforms is rooted in their distinct algorithmic mechanisms and content ecosystems. Douyin's recommendation algorithm prioritizes high-engagement content, forming a positive feedback loop of “interaction-exposure-more interaction,” which explains its higher likes, comments and shares. This platform feature also lowers the exposure threshold for high-quality professional content, which is consistent with the higher proportion of professional healthcare creators (55%) on Douyin and its overall higher quality scores.

In contrast, Bilibili's content distribution relies more on user interest tags and fan followings, with weaker algorithmic push for vertical medical content. Meanwhile, the proportion of professional healthcare creators on Bilibili is only 36%, and patient experience and non-professional science content account for a higher share, which may explain why longer video duration does not translate into higher overall quality scores. A plausible contributing factor is that Douyin has a stricter professional identity verification mechanism for medical creators, which raises the baseline of content authority, while Bilibili's access threshold for medical content uploaders is relatively loose, further amplifying the quality difference between the two platforms. It should be noted that this study did not directly verify the causal relationship between verification policies and content quality, and this inference remains to be confirmed by targeted studies.

### Comparison of uploader differences

Videos published by platform-certified professional healthcare providers performed best in terms of scientific rigor, accuracy, and comprehensiveness in our sample. Most of these videos were compiled in accordance with current clinical guidelines, adopting a clear logical framework covering etiology, clinical staging, treatment and daily nursing, and interpreting professional medical knowledge in easy-to-understand language. For example, videos posted by dermatologists provide detailed explanations of treatment protocols for different RTOG grades of radiation dermatitis, using clinical cases to illustrate the timing and dosage of medications. Some videos also demonstrate practical procedures, such as the application of wound healing agents and dressing change protocols.

By comparison, content released by non-profit institutions is also authoritative with adequate evidence and diversified presentation forms such as animation and expert interviews. Nevertheless, most institutional videos lack targeted personalized guidance for radiotherapy patients in China, which reduces their practical value for local audiences.

Individual-generated content can be split into two types: patient sharing videos deliver emotional comfort but lack standardized clinical evidence, so their care suggestions are limited to personal individual experience and cannot be universally applied; health blogger videos pursue attractive presentation and high dissemination potential, yet many contain oversimplified statements or unvalidated folk remedies that may mislead radiotherapy patients, consistent with the lower mDISCERN and JAMA scores we observed among individual uploaders. Importantly, professional medical creators only accounted for 47.37% of all included videos, and merely 36% on Bilibili, meaning non-professional content still occupies the majority of RD-related short videos and creates risks of inaccurate health information dissemination.

### Video content

A thematic analysis of 190 valid videos across the two major platforms revealed that videos related to radiation dermatitis covered a wide range of topics, with significant differences in topic distribution between the platforms (*p* < 0.05): Both Douyin and Bilibili focused primarily on treatment methods (61/190, 32%); on Douyin, news reports (8/117, 6.8%) had the lowest proportion, while on Bilibili, preventive measures (3/73, 4.1%) had the lowest proportion. Most doctors mentioned various RD treatment methods but did not specify which treatment was more suitable for which stage, which could lead to a narrow understanding of the disease's treatment among patients. Patients may tend to adopt methods perceived as more effective or promising better outcomes rather than treatments suited to their individual conditions. Most doctors also overlooked the risk factors for controlling RD.

Regarding preventive measures, Douyin videos often emphasize “simplicity and ease of use,” such as “wearing loose-fitting cotton clothing during radiation therapy” or “gently washing the irradiated area with warm water.” In contrast, Bilibili videos delve deeper into the underlying principles, such as “loose clothing reduces friction damage, and cotton materials are breathable, preventing irritation from sweat.” Some videos also incorporate anatomical and physiological knowledge to explain the importance of skin protection. Regarding Traditional Chinese Medicine (TCM) content, herbal treatments account for the largest share. Videos posted by doctors introduce the appropriate scenarios and usage methods for topical TCM treatments—such as Compound Lithospermum Oil and Ulcer Oil—based on various pathogenic mechanisms, emphasizing the importance of syndrome differentiation and treatment to avoid blind recommendations.

It is worth noting that some videos suffer from “over-simplification” or “one-sided interpretations.” For example, regarding high-temperature stimulation, some videos merely state “avoid high-temperature stimulation” without specifying the intensity of the stimulation, duration, or precautions. Such one-sidedness may lead to misunderstandings among users, which in turn could affect the effectiveness of care.

### Video quality

In terms of video quality, Douyin videos obtained numerically higher median GQS scores than Bilibili, but the difference lacked statistical significance (*P* = 0.148). Meanwhile, Douyin showed significantly higher mDISCERN and JAMA scores (both *P* < 0.05). A plausible explanation for this difference is that stricter professional identity verification mechanisms for healthcare creators on the platform help improve the transparency of author qualifications, and professional uploaders tend to present the pros and cons of different treatment options more objectively. It should be noted that this study did not directly examine creator verification status as a predictor of video quality, and the association between platform governance policies and content quality remains to be verified by further targeted studies. Further analysis shows that videos uploaded by professional healthcare providers score significantly higher in terms of likes, comments, GQS, JAMA, and mDISCERN scores compared to videos uploaded by other users. This indicates that videos produced by professional healthcare providers have advantages in content quality, information reliability, and audience recognition. These videos typically contain more specialized medical knowledge, detailed explanations, and scientific evidence, making them more popular and trusted by viewers.

### Correlation analysis

This study found a strong positive correlation (greater than 0.78) among likes, comments, saves, and shares. This implies that content receiving a high number of likes is also more likely to receive more comments, be saved by more users, and be shared. This high level of correlation suggests that positive user feedback often manifests in multiple forms of engagement. Additionally, there is a strong correlation between video quality scores and engagement metrics. The correlation coefficient between the mDISCERN score and the number of likes was 0.89 (*p* < 0.05); with the number of saves, it was 0.86 (*p* < 0.05); and with the number of shares, it was 0.85 (*p* < 0.05). These results are consistent with previous studies. However, unlike mDISCERN, JAMA scores showed weak and non-significant correlations with audience engagement indicators. This may be because JAMA primarily evaluates the reliability, transparency, and credibility of medical information, whereas user engagement on short-video platforms is influenced by multiple factors, including presentation style, emotional appeal, topic popularity, and platform recommendation mechanisms. Therefore, scientifically rigorous content may not always receive higher levels of audience interaction. This finding suggests that information quality and communication effectiveness represent related but distinct dimensions of online health education.

Correlation analysis indicates that higher alignment between content themes and user needs is associated with higher-quality engagement and greater practical value for viewers. For example, videos focusing on scenario-specific topics such as “radiation dermatitis care for head and neck radiotherapy” achieved substantially higher save rates relative to the average, despite not having the highest like counts, reflecting strong practical demand for targeted content among the audience. Conversely, videos with overly broad and non-specific themes tend to have lower user utility even with high raw interaction volumes, as shown by their lower save-to-like and share-to-like ratios.

### Practical significance

This study focuses on radiation dermatitis, a common clinical condition. The study utilized three internationally recognized tools—GQS, JAMA, and mDISCERN—to quantitatively evaluate video quality. Additionally, it validated the association between engagement metrics and content quality through correlation analysis, thereby providing empirical evidence to optimize health education related to clinical treatment.

Short-form video platforms cater to the public demand for convenient, on-demand access to health information, helping patients grasp core treatment objectives and key care procedures, and may support improved adherence to clinically prescribed care regimens (33).

Our analysis shows that treatment-focused educational content uploaded by professional healthcare providers generally conforms to the core framework of mainstream clinical guidelines, delivering standardized skin care and pharmacotherapy information in an accessible format, which is consistent with prior quality assessments of radiotherapy-related short videos on the same platforms (34). Short-form videos expose patients to information on standard management strategies for radiation dermatitis, including the use of topical corticosteroids, topical herbal preparations, and basic wound care principles. This may help patients identify key care points and reduce exposure to unsubstantiated or misleading health claims, and may serve as a supplementary information resource for populations with limited access to face-to-face clinical consultations, such as rural residents and older adults, aligning with established evidence on social media's role in dermatology patient education (35).

Additionally, this study provides an objective snapshot of the dissemination patterns of Western medicine and traditional Chinese medicine (TCM) content across short-video platforms. Content covering TCM modalities such as acupuncture and acupoint plaster application is present in the sampled videos, alongside content addressing Western pharmacological and non-pharmacological care. This finding illustrates the heterogeneous nature of health information on social media platforms, and may inform the design of interdisciplinary health education resources by clinical practitioners, in line with best practices for patient education in radiation oncology settings (36, 37).

### Limitations

This study has several critical limitations. First, all videos were collected on November 1, 2025 using a fixed set of search keywords. As a cross-sectional analysis, it only captures a static snapshot of platform content, which evolves continuously over time. Single-time-point sampling leads to temporal algorithm bias, and repeated sampling at multiple staggered time points may yield divergent statistical results. Second, restricted by the open data access policies of Douyin and Bilibili, precise view counts and dislike metrics were unavailable. Without view data, we cannot distinguish widely disseminated high-quality educational videos from niche clips with elevated local engagement. The absence of negative feedback data tends to overestimate audience approval unilaterally. Furthermore, high volumes of likes, collections and shares often stem from sensational clickbait headlines rather than rigorous medical information, confounding the correlation analysis between audience interaction and content quality. Third, this cross-sectional observational study can only identify correlational associations rather than causal relationships. All platform governance suggestions proposed herein are preliminary inferences drawn solely from the current sample and cannot be regarded as universal, definitive solutions. Finally, this study relied on independent scoring by two licensed physicians, and while inter-rater reliability was verified using Cohen's kappa coefficient, the influence of subjective judgment could not be entirely avoided. Different evaluators may have varying interpretations of the scientific rigor and comprehensiveness of the same video.

Future research may expand sample sizes, include more video platforms, integrate multidimensional evaluation indicators, adopt artificial intelligence-assisted assessment approaches, and conduct cross-linguistic and cross-cultural comparative studies. Here, cross-linguistic and cross-cultural comparative studies refer to comparative analyses of medical science communication videos from different languages and cultural backgrounds (e.g., Chinese, English, Spanish, Arabic, Japanese, and Russian) on domestic and international short-video platforms, focusing on differences in content quality, presentation styles, and audience engagement patterns.

## Conclusion

This study identified characteristics associated with higher-quality radiation dermatitis-related videos, helping them understand the current state of online videos related to radiation dermatitis on social media platforms. These findings are beneficial to the public, content creators, and platforms alike. The study evaluated the quality, dissemination characteristics, and reliability of videos related to radiation dermatitis on Douyin and Bilibili. The results indicate that professional creators produce higher.

quality content on average, but they are not the majority source; their videos significantly outperform those of other uploaders in terms of scientific accuracy and engagement levels, and there is a strong positive correlation between video quality and engagement metrics. The platform management recommendations proposed in this paper are only reference opinions based on observed associations from this single cross-sectional survey, instead of absolute, definitive solutions. The two platforms have distinct characteristics: Douyin videos are shorter and more interactive, while Bilibili focuses on in-depth analysis. However, some videos suffer from issues such as one-sided interpretations and a lack of personalized guidance, highlighting the need for further improvements in video quality and the demand for more high-quality short videos related to radiation dermatitis. Additionally, platforms should strengthen oversight and optimize algorithms to encourage professional healthcare providers to publish more accurate, needs-based educational videos. At the same time, creators need to balance content conciseness with depth to help the public access standardized information on the care and treatment of radiation dermatitis.

## Data Availability

The raw data supporting the conclusions of this article will be made available by the authors, without undue reservation.
