# Supplementary material for: Evaluating the quality and reliability of radiation dermatitis related videos on Douyin and Bilibili: a cross-sectional analysis
Source: Front Public Health. 2026 Jul 27;14:1851530. doi: 10.3389/fpubh.2026.1851530 (PMC13453699; doi:10.3389/fpubh.2026.1851530)
Supplement: Supplementary file 1 [file Table_1.doc]

**Supplementary Table 1 The Global Quality Score (GQS) instrument for evaluating the quality about videos, scale ranging from 1 (poor quality) to 5 (excellent quality)**

| **Score** | **GQS Definition** | **Description** |
| --- | --- | --- |
| 1 | Poor quality | Poor quality, poor flow of the video, most information missing, not at all useful for patients. |
| 2 | Generally poor quality | Generally poor quality and poor flow, some information listed but many important topics missing, of very limited use to patients. |
| 3 | Moderate quality | Moderate quality, some important information is adequately discussed. |
| 4 | Good quality | Good quality good flow, most relevant information is covered, useful for patients. |
| 5 | Excellent quality | Excellent quality and flow, very useful for patients. |

**Supplementary Table 2.** The Journal of American Medical Association (JAMA) benchmark criteria.

| **Criteria**  **(1 point for each)** | **Description** |
| --- | --- |
| Authorship | Author and contributor credentials and their affiliations should be provided |
| Attribution | Clearly lists all copyright information and states references and sources for content |
| Currency | Initial date of posted content and subsequent updates to content should be provided |
| Disclosure | Conflicts of interest, funding, sponsorship, advertising, support, and video ownership should be fully disclosed |

**Supplementary Table 3** The modified DISCERN score for evaluating the reliability about videos (score accumulation, a positive answer accumulates one point)

| **No.** | **Question** |
| --- | --- |
| 1 | Are the aims clear? |
| 2 | Does it achieve its aims? |
| 3 | Is it relevant? |
| 4 | Is it clear what sources of information were used to compile the publication (other than the author or producer)? |
| 5 | Is it clear when the information used or reported in the publication was produced? |

**Supplementary Table 4** Definitions and classification criteria for uploader categories

| **Category level** | **Category name** | **Definition** | **Judgment criteria** |
| --- | --- | --- | --- |
| Detailed subgroups (7 categories) | Dermatologists | Clinicians with dermatology practice qualifications who release RD-related professional content | Account has official medical certification; profile clearly states dermatologist identity; content is delivered from a clinical dermatology perspective |
|  | Non-dermatologists | Clinicians of other specialties (oncology, nursing, etc.) who release RD-related content | Account has official medical certification; profile clearly states non-dermatology clinical background; content is delivered from a clinical |
|  | Science communicators | Non-medical practitioners engaged in health science popularization with no clinical practice qualifications | No official medical certification; profile positions itself as a health popular science blogger; content focuses on popularizing basic medical knowledge |
|  | Non-profit institutions | Accounts of public hospitals, academic organizations, non-profit medical institutions | Account subject is a public welfare institution; account name includes official institution name; no obvious commercial promotion content |
|  | Profit-making institutions | Accounts of private medical institutions, pharmaceutical companies, medical beauty institutions | Account subject is a commercial organization; content contains obvious product or service promotion information |
|  | Patients | Accounts that share personal radiotherapy and RD treatment experience from a patient perspective | Content is based on first-person personal disease experience; no professional medical identity statement |
|  | Individual users | Ordinary users who release relevant content without clear identity positioning | No clear professional or patient identity label; content is simple reposting, opinion expression or life sharing |
| Merged macro-categories (4 categories) | Professional healthcare providers | Macro-category aggregated from clinical practitioners of all specialties | Aggregated from: Dermatologists and Non-dermatologists |
|  | Institutions | Macro-category aggregated from all organizational accounts | Aggregated from: Non-profit institutions and Profit-making institutions |
|  | Non-professional healthcare providers | Macro-category for non-qualified health communicators | Aggregated from: Science communicators |
|  | Individual Users | Macro-category for personal accounts without professional positioning | Aggregated from: Patients and General individual users |
| Ambiguous sample rule | — | — | Two reviewers independently coded all samples; samples with inconsistent classification were discussed collectively. If no consensus was reached, the final category was determined by the corresponding author. |

**Supplementary Table 5** Definitions and coding rules for video content categories

| **Category name** | **Definition** | **Inclusion scope** | **Multi-theme coding rule** |
| --- | --- | --- | --- |
| Clinical manifestations | Content focusing on symptoms, signs and staging of radiation dermatitis | RTOG grading introduction, symptom description of different degrees of RD, picture display of skin lesions, clinical case presentation | Dominant theme principle: each video is assigned to only 1 category, subject to the core theme of the video and the content accounting for ≥50% of the total duration. |
| Etiology and Predisposing Factors | Content explaining the pathogenesis and influencing factors of RD | Mechanism of radiation skin injury, analysis of high-risk factors for severe RD, explanation of population susceptibility | For videos with multiple evenly distributed themes (no single theme accounts for more than 50%), two coders independently judged and reached a consensus through negotiation. |
| News reports | Content in the form of news, policy interpretation or event release | Release of new clinical guidelines, reporting of relevant academic | No multi-label classification is adopted to ensure the mutual exclusivity of statistical analysis among groups. |
| Patient experiences or others | Personal experience sharing and other unclassified content | Patient treatment diary, experience sharing of side effect management, life vlog related to radiotherapy, simple reposting content |  |
| Preventive measures | Content focusing on RD prevention and daily skin care | Skin care recommendations before and during radiotherapy, daily precautions, risk avoidance guidance |  |
| Scientific introduction | Popular science content explaining basic concepts of radiotherapy and RD | Basic knowledge of radiotherapy, popularization of RD disease concepts, explanation of common misunderstandings |  |
| Therapeutic Approaches | Content introducing treatment plans and intervention methods for RD | Western medicine treatment (topical drugs, systemic medication), traditional Chinese medicine intervention, physical therapy, nursing operation demonstration |  |
